# First person – Kishalay Ghosh and Rohit Iyer

**DOI:** 10.1242/bio.062123

**Published:** 2025-07-25

## Abstract

First Person is a series of interviews with the first authors of a selection of papers published in Biology Open, helping researchers promote themselves alongside their papers. Kishalay Ghosh and Rohit Iyer are co-first authors on ‘ Genetic perturbation of cellular homeostasis regulates integrated stress response signaling to control *Drosophila* hematopoiesis’, published in BiO. Kishalay conducted the research described in this article while a project associate in Dr Rohan J. Khadilkar's lab at the Advanced Centre for Treatment, Research and Education in Cancer (ACTREC), Tata Memorial Centre, Navi Mumbai, India. He is now a PhD student in the lab of Jaime De Juan-Sanz at ICM Paris Brain Institute, Hôpital Pitié, Paris, France, investigating metabolic regulation of neuronal circuit function and behaviour. Rohit is a project associate in the lab of Dr Rohan J. Khadilkar at ACTREC, India, investigating understanding the intricacies of host-pathogen interactions.


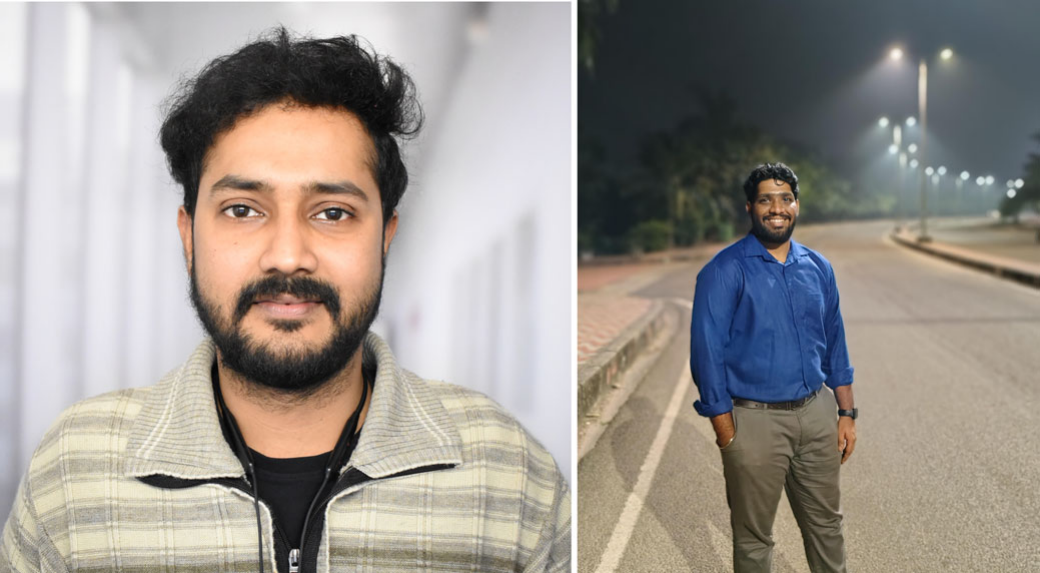


**Kishalay Ghosh and Rohit Iyer**

**Describe your scientific journey and your current research focus**

**KG:** My scientific training followed a diverse trajectory, which started with an undergraduate degree in plant sciences from Shantiniketan, followed by a master's degree in biotechnology from IIT, an esteemed technical university in India. After a few internships in immunology and neuroscience labs, I chose to understand the nuances of cell signalling through developmental biology and genetics, which led to this beautiful collaboration with Dr Rohan Khadilkar in the stem cell and tissue homeostasis lab at ACTREC between 2021-24, where I worked as a research associate. I am currently working on understanding the role of mitochondrial metabolism in neuronal circuit function at ICM- Paris Brain Institute with Dr Jaime De Juan Sanz for my PhD in Paris.

**RKI:** On the other hand, I have had a very streamlined trajectory, starting with my higher secondary education, where I was introduced to biotechnology for the first time, which I found so interesting and fascinating that I decided to pursue it later through an MTech integrated degree in biotechnology. Later, I got interested in immunology and joined the stem cell and tissue homeostasis lab at ACTREC, from 2023 to the present day, under the supervision and support of Dr Rohan Khadilkar, working on understanding the intricacies of cellular signalling during *Drosophila* haematopoiesis. While working on blood cell homeostasis under stress, what intrigued us was the heterogeneity within the blood progenitor population, which, to our understanding, makes the system remarkably flexible in its response to stress. These progenitors differentiate through intrinsic and extrinsic local stresses originating within the hematopoietic compartment or the systemic stresses induced in another part of the body. It is fascinating to observe how the fine balance between the progenitors and the differentiated cell pool gets perturbed in response to various stresses. These stressors also make the progenitors biased towards one of the lineages over the others during differentiation. Interestingly, different stressors skew the relative abundance of blood cell types in different directions. A part of the lab's current interest is to understand the flexibility and heterogeneity of progenitors at the cellular level, which I believe can unfold the molecular underpinning of these lineage biases in their stress response.

**Who or what inspired you to become a scientist?**

**KG:** Understanding how any system works gives me joy. It can be from the faraway galaxies or the economic and political machinery of a country, to the chemical homeostasis of thousands of different molecules inside a living cell. The pleasure of solving puzzles and unravelling the truths led me to science. As I made my way into this profession, I realised that the critical thinking you develop through your profession also enlightens you beyond your profession, making your perspective widen and your observations sharpen towards every aspect of your life. This is the profession where you have the intellectual freedom and autonomy of artists, and the stability and security of a mainstream white-collar job. Thus, I get thrilled to see my older self, 25 years down the line with a bunch of younger colleagues and share the same cognitive flexibility and enthusiasm. If I have to name a single person who particularly inspired me greatly, it would be Prof. Susumu Tonegawa. His tendency to combine knowledge from different fields led to great discoveries throughout the last three decades of the 1900s and the first decade and a half of this century in immunology and systems neuroscience.

**RKI:** Well, I'm not a scientist yet. But yes, I think I'm on the right track to becoming one. I have always been an inquisitive person since the beginning, and have always been questioning and seeking answers. I would find some phenomenon or concept interesting, and while trying to read and understand about it, I would often dive very deep in no time and explore the concept's connections and crosstalk with other stuff. At the end of the session, I would have gathered a great deal of knowledge and begun to think critically about it. Overall, I would enjoy learning and understanding new theories and concepts, but I feel there can be better streamlining in my process of learning for the efficient use of time and effort. I would attribute all these qualities to Dr A.P.J. Abdul Kalam, the missile man of India and former Indian President, who actually inspired me the most to pursue science. He once rightfully said, and I quote, “Learning gives creativity, creativity leads to thinking, thinking provides knowledge, and knowledge makes you great”. I truly believe in his words and would like to follow them for the rest of my life.

**How would you explain the main finding of your paper?**

**KG and RKI:** We shed 600,000 skin cells daily, and it accounts for 680 grams per year. However, we have skin stem cells beneath our skin, which divide and give rise to new skin cells, compensating for the loss of old ones. This ensures that our skin cell count is maintained and its functions remain intact. Thus, a homeostatic balance exists between the stem cells and the terminally differentiated cells, which is kept under normal conditions. A similar process happens with a number of tissues in our body where stem cells or progenitor cells are reserved in those tissues, and their function is to replace different types of old, dysfunctional differentiated cells of that tissue by producing new cells. Thus, any dysregulation in these cells either results in overproduction of new cells and subsequent formation of cancer, or inadequate division of them results in tissue degeneration. We work on some of these progenitor cell types in our lab using *Drosophila* (fruit fly) as a model organism. As the hematopoietic stem cells reside in our bone marrow, which continue to supply fresh blood stem cells throughout our lifetime, fruit fly larvae also have an organ called the lymph gland that houses the hematopoietic progenitors and the subsequently differentiated hemocytes, too. In this study, we induced the stress of inflammation in different cell subsets of the lymph gland to see how these progenitor cells respond to the physiological stress. What we observe is that, under these stressful conditions, the homeostatic balance maintained between the progenitors and differentiated haemocyte cell pool gets disrupted and leans more towards differentiation. Also, some stress-responsive signalling pathways, like integrated stress response (ISR), get activated in those cells to help them adapt and counter the stress in order to recoup and survive. Overall, this project gives good insights about how perturbation of cellular homeostasis in the lymph gland can cause defects in haematopoiesis.

**What are the potential implications of this finding for your field of research?**

**KG:** As we grow old, our stem cell functions decline. An aging body produces harmful substances that put our stem cells under chronic stress. The aging of blood stem cells leads to the production of poorly functional blood cells, which is associated with the higher incidence of diseases like haematological neoplasms, rheumatoid arthritis, and cardiovascular malfunction. Uncontrolled division of blood stem cells is the cause of higher rates of several blood cancers among the older population. Our research indicates that if you can activate the ISR signalling inside the stem cells, it can rescue the harmful effects of age-associated cellular stress on the stem cells.

**RKI:** This study aims to uncover the effects of modulating the molecular circuitry of cellular homeostasis in the *Drosophila* lymph gland and understand its cell-intrinsic and extrinsic regulation and how its perturbation in both genetic and chemical manner can impact haematopoiesis. Our study suggests that ISR signalling is activated under conditions of constitutively activated NF-κB pathways (Imd and Toll), and ISR perturbation indicated the homeostatic balance inclining more towards haemocyte differentiation. In addition, ectopic over-expression of ISR effectors in the genetic background of hyperactivated Imd could rescue the defects in haematopoiesis. Overall, our study shows that modulating cellular homeostasis in a localised or systemic manner impacts the lymph gland haematopoiesis. Moreover, ISR signalling has an important role in countering stress related to inflammation and oxidative stress and in regulating blood cell homeostasis in *Drosophila*.

It felt like all the late nights setting up crosses and optimizing those protocols had finally paid off

**Figure:**
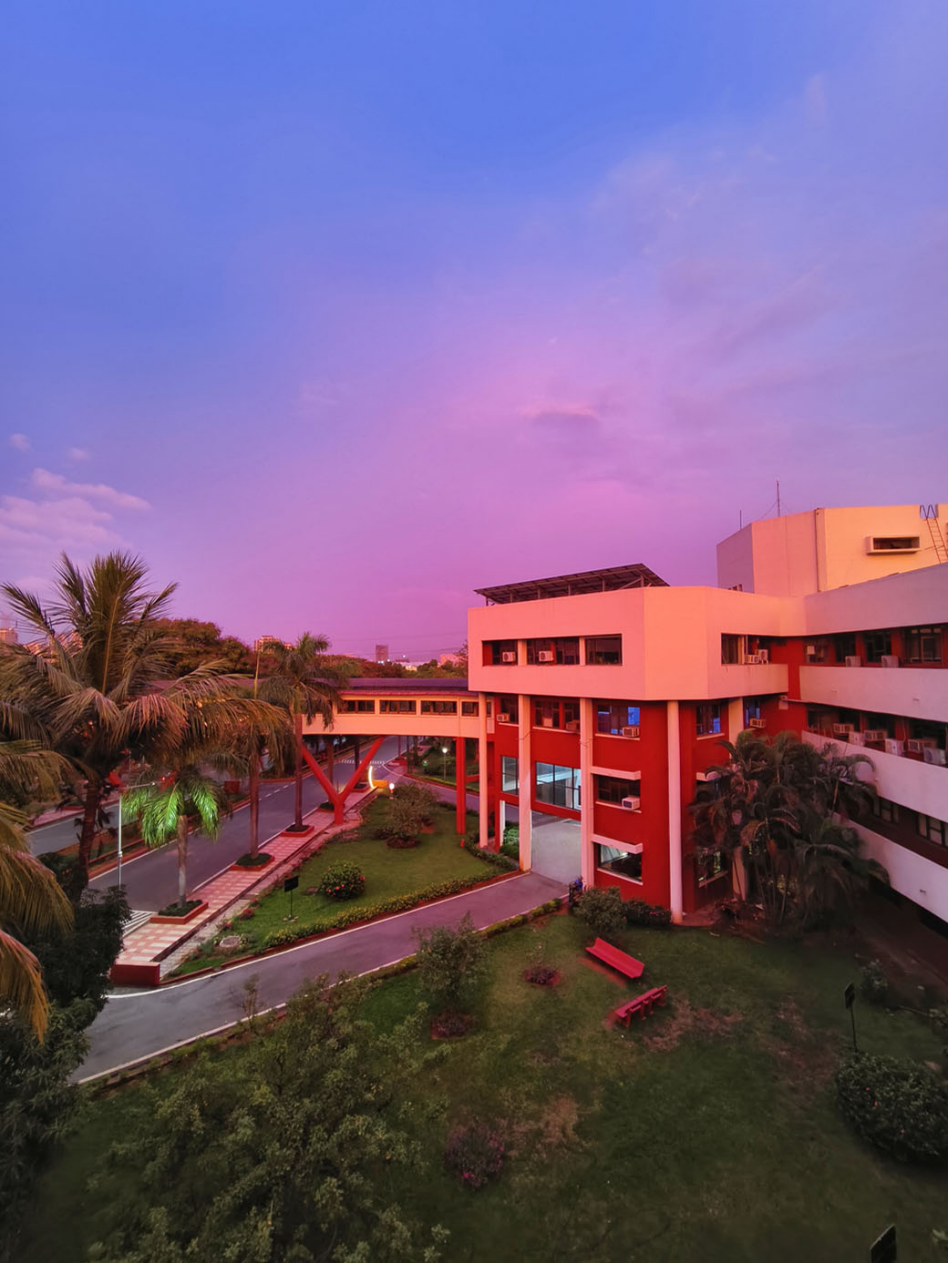
Golden hour hues before sunset at Cancer Research Institute, ACTREC – Tata Memorial Centre, Navi Mumbai, India.

**Which part of this research project was the most rewarding?**

**KG:** One of the most rewarding parts of this project was discovering that activating the ISR pathway could actually reverse the effects of chronic inflammation in the lymph gland. We had been seeing aberrant hematopoisis with Toll and Imd overactivation, and it honestly felt like the system was too stressed to recover. Moreover, we had one criticism whenever we presented results from our project in some conference, which is whether the effect of our genetic model of cellular stress, like increased differentiation of immune cells from blood progenitors, is a result of chronic inflammatory stress or just a matter of direct immune activation. But when we introduced ISR components like Gcn2 or used chemical activators like Histidinol, we started seeing a rescue in differentiation patterns. It not only confirmed that the catastrophic effect of Toll or IMD pathway activation is not just because of immune activation but is a chronic inflammatory insult on blood progenitors. Our data clearly showed that even under chronic stress, the system had a way to bounce back. It was a big relief! It felt like all the late nights setting up crosses and optimizing those protocols had finally paid off. It is delightful to see that our research on cute fruit flies can answer big questions and propose potential solutions in the field of aging and age-associated haematological malignancies.

**RKI:** The fly maintenance work involving rigorous screening of pupae for sexual dimorphism and screening of larvae using reporter expression like GFP/RFP, setting up multi-generational genetic crosses as a prerequisite for experiments to follow were at times cumbersome and challenging, but extremely rewarding at the end when we could uncover the mechanisms underlying homeostasis using these genetic epistasis experiments. Additionally, presenting the lab's work at a national conference on cell biology via oral presentation in front of a big audience composed of students, professors, and big-shot scientists from the field and getting inputs and appreciation from them was also most rewarding.

**What do you enjoy most about being an early-career researcher?**

**KG:** The best part of being an early-career researcher is getting guidance and supervision from scientists, including team leaders who are not only more knowledgeable but have already lived and thrived in the life I am currently living. I consider myself lucky in this regard, as I have worked with some great PIs so far who understood my strengths and limitations and offered me personalised mentorships. As we are progressing in our careers, getting to learning new techniques and developing soft skills is kind of exciting, as every passing year we grow exponentially and rediscover ourselves in the process.

**RKI:** I have a long way to go as an established researcher and exploring what's in store for me along the journey seems very exciting. Being an early-stage researcher is like standing at the edge of a forest where previously others have used a compass and map to show how best to traverse it. I'm able to roam at will, take unexpected paths, and also have the privilege of getting lost a little. As a research associate in the lab, I have already seen the hardships faced by my fellow PhD colleagues and have already started reading the rulebook of pursuing a PhD.

**What piece of advice would you give to the next generation of researchers?**

**KG:** The first thing I can tell the next generation of researchers is that basics are important; you should have habits of reading textbooks in your undergraduate studies, because nothing can be understood in isolation. For example, even if you are working in the system biology, you need to have a decent understanding of fundamentals of cell biology and structural biology; and easiest way to ensure that is to study passionately in your undergrad because once you become specialised in a particular field, it's much more difficult to go back and fill in the gaps in a topic outside of your field of research. I assume that we all came to research because we had a very flowery idea about what this felt like, i.e. doing cool experiments, producing exciting research, and making groundbreaking discoveries now and then. But the actual picture is a bit different, where we repeat the same set of experiments again and again, most of the results are negative, groundbreaking discoveries happen in a ‘once in a lifetime’ frequency, that too, only if you are super smart and exceptionally lucky at the same time. Thus, in my opinion, you should not focus on the outcomes but celebrate the growth it brings to you through this lifestyle.

**RKI:** A small piece of advice is that one has to remain positive, motivated, and persistent at all times, as situations can be very uncertain and hard work alone sometimes may not suffice at that time. You should see life as it is and just go with the flow and not get disheartened when experiments fail. Instead, try to perceive the positive side of how you can troubleshoot and identify the core of problems, thereby never repeating them again and propagating your lessons to others, too. Also, work-life balance is a must for a healthy mind. Make sure you reward yourself with whatever you like doing at the end of the day for a peaceful sleep.

**What's next for you?**

**KG:** I am now in Paris, and I've started a PhD in neuroscience at ICM Paris Brain Institute with Dr Jaime De Juan Sanz, and I've been working on developing mitochondria-targeted optogenetic tools to study the causal relationship between mitochondrial bioenergetics and synaptic transmission since October 2024.

**RKI:** I'm now actively looking and applying for PhD positions in Europe. My research interests are inclined towards molecular parasitology, genetics, stem cell biology and even exploring other model organisms like zebrafish, etc., in the context of developmental biology.
